# Internally controlled RNA sequencing comparisons using nucleoside recoding chemistry

**DOI:** 10.1093/nar/gkac693

**Published:** 2022-08-26

**Authors:** Meaghan C S Courvan, Rachel O Niederer, Isaac W Vock, Lea Kiefer, Wendy V Gilbert, Matthew D Simon

**Affiliations:** Department of Molecular Biophysics and Biochemistry, Yale University, New Haven, CT06536, USA; Institute of Biomolecular Design and Discovery, Yale University, West Haven, CT06477, USA; Department of Molecular Biophysics and Biochemistry, Yale University, New Haven, CT06536, USA; Department of Molecular Biophysics and Biochemistry, Yale University, New Haven, CT06536, USA; Institute of Biomolecular Design and Discovery, Yale University, West Haven, CT06477, USA; Department of Molecular Biophysics and Biochemistry, Yale University, New Haven, CT06536, USA; Institute of Biomolecular Design and Discovery, Yale University, West Haven, CT06477, USA; Department of Molecular Biophysics and Biochemistry, Yale University, New Haven, CT06536, USA; Department of Molecular Biophysics and Biochemistry, Yale University, New Haven, CT06536, USA; Institute of Biomolecular Design and Discovery, Yale University, West Haven, CT06477, USA

## Abstract

Quantitative comparisons of RNA levels from different samples can lead to new biological understanding if they are able to distinguish biological variation from variable sample preparation. These challenges are pronounced in comparisons that require complex biochemical manipulations (e.g. isolating polysomes to study translation). Here, we present Transcript Regulation Identified by Labeling with Nucleoside Analogues in Cell Culture (TILAC), an internally controlled approach for quantitative comparisons of RNA content. TILAC uses two metabolic labels, 4-thiouridine (s^4^U) and 6-thioguanosine (s^6^G), to differentially label RNAs in cells, allowing experimental and control samples to be pooled prior to downstream biochemical manipulations. TILAC leverages nucleoside recoding chemistry to generate characteristic sequencing signatures for each label and uses statistical modeling to compare the abundance of RNA transcripts between samples. We verified the performance of TILAC in transcriptome-scale experiments involving RNA polymerase II inhibition and heat shock. We then applied TILAC to quantify changes in mRNA association with actively translating ribosomes during sodium arsenite stress and discovered a set of transcripts that are translationally upregulated, including *MCM2* and *DDX5*. TILAC is broadly applicable to uncover differences between samples leading to improved biological insights.

## INTRODUCTION

The differences in RNA levels between experimental conditions can provide an understanding of the underlying biology, but accurate assessment of these differences requires the real biological variation to be distinguished from technical variation (1). Biochemical enrichment (e.g. polysome isolation) often requires a number of sensitive steps that are challenging to perform identically on multiple samples. During the sequencing process, RNA isolation, shearing, adaptor ligation, and PCR amplification can also introduce biases (2–4). Furthermore, analyses of traditional RNA sequencing experiments must include robust across-sample normalization procedures while also accounting for several sources of experimental variance (i.e. heterodispersivity in read counts, uneven read coverage, etc.) (5,6). These challenges limit the accuracy of biological conclusions made when comparing RNA-seq data between samples; many of these challenges could be avoided if samples experienced the exact same handling throughout an experiment.

One approach to increase the accuracy of comparisons made by RNA-seq is to increase the number of replicate samples that are sequenced and analysed (7). Unfortunately, the resources required for high replicate experimental designs (with six or more replicates) can be prohibitive. Statistical modelling can instead be used to improve estimates from experiments with low numbers of replicates (5,8). While these methods have proven indispensable when accounting for the technical variation observed in RNA-seq data, they do not compensate for the loss of statistical power caused by experimental steps that introduce large variation between replicates (5,8). Ideally, it would be possible to control for this variation by handling the experimental and control samples identically during biochemical manipulations.

Normalizing read counts between samples is an additional challenge when using traditional RNA-seq based analyses. Specifically, many methods require the assumption that the total RNA content is equal across samples and that a minority of RNA transcripts exhibit significantly different levels between conditions. This assumption is problematic when comparing samples that exhibit large differences in total RNA content, such as when examining cells treated with an inhibitor of transcription or those that are genetically depleted of a broad-acting transcription factor (2,9,10). To address this issue, exogenous “spike-in” RNA can be added to all samples to serve as a standard for normalization (11,12). While this can be an effective remedy, normalization methods that do not require spike-ins are possible (13), and we designed an alternative approach to RNA-seq that is internally controlled and does not require spike-in normalization.

We were inspired by proteomic experiments developed for internally controlled analysis of protein levels. SILAC (Stable Isotope Labelling by Amino Acids in Cell Culture) is a gold standard method for comparing protein levels between two samples that have undergone complex biochemical manipulations such as fractionation (14–16). In SILAC, cells from the condition of interest and a control condition are grown in different media; one condition is grown in the presence of heavy-isotope amino acids, and the other with light-isotope amino acids. The different labels get incorporated into proteins and thereby mark the sample of origin for each peptide in mass spectrometry experiments. This *in vivo* incorporation of distinct labels allows the samples to be mixed and handled identically throughout the experiment.

Here we describe the development of an analogous approach to compare RNA levels, called Transcript Regulation Identified by Labelling with Nucleoside Analogues in Cell Culture (TILAC). We reasoned it would be possible to use s^4^U and s^6^G as distinct metabolic labels to distinguish two different RNA samples. Thanks to recent developments in nucleoside recoding chemistry (17–22), including TimeLapse chemistry (21,22), the thiolated nucleosides s^4^U and s^6^G can be chemically recoded to have the same hydrogen bonding patterns as those of a cytidine (C) or adenosine (A), respectively. These recoded nucleosides are observed as specific T-to-C or G-to-A mutations in sequencing experiments, and we therefore reasoned that these mutations could be used to indicate the sample of origin for sequencing reads. Comparing the levels of T-to-C mutations and G-to-A mutations in reads that map to each transcript would enable quantification of different RNA levels between samples.

We first established the feasibility of TILAC using simulations. We next verified that TILAC can reveal changes in RNA levels between samples without fractionation, including in cases where there are global changes in transcription. Finally, we used TILAC to study RNA levels in different samples after fractionation. Specifically, we examined translational regulation upon cellular stress and discovered polysome enrichment of transcripts that encode helicases, including the DDX5 and MCM2 helicases that are known to play a role in stress granule resolution. This discovery provides new insight into translational control during stress and demonstrates the power of TILAC to uncover previously unobserved biological regulation.

## MATERIALS AND METHODS

### Tissue culture and metabolic labelling

*Drosophila* S2 cells were cultured in Schneider medium (Lonza) supplemented with 10% heat-inactivated FBS (Invitrogen) and 1% penicillin–streptomycin (Millipore) and maintained at 27°C. Cultures were split every 3 days to a concentration of 5 × 10^5^ cells/ml. HEK293T cells were grown at 37°C in DMEM media (Invitrogen, high glucose) supplemented with 10% FBS (Invitrogen) and 1% penicillin–streptomycin (Millipore). Cells were split when they reached 70% confluence.

To prepare stock solutions of the nucleoside analogues, s^4^U was dissolved in water and s^6^G was dissolved in DMSO. To control for any effects of the water or DMSO, s^4^U samples were also treated with an equivalent volume of DMSO, and s^6^G samples were also treated with an equivalent volume of water.

### Quantifying viability

HEK293T cells were grown at 37°C in DMEM supplemented with 10% FBS and 1% penicillin–streptomycin. Cells were plated at 10^6^ cells/ml in a 96-well microtiter plate. The next day, cells were treated with the indicated amount of nucleoside analogue, control, or 1% triton to induce cell death. Because s^4^U is dissolved in water, it is compared to a water-only control. Because s^6^G is dissolved in DMSO, it is compared to a DMSO-only control. Cell viability was assessed using the MTT Cell Proliferation Assay (ATCC) according to the manufacturer's instructions.

### Heat shock

*Drosophila* S2 cells were grown in 6-well plates. For heat shock treatments, cells were incubated at 37°C for 1 h and treated with 100 μM nucleoside analogue (s^4^U or s^6^G—purchased from Alfa Aesar and Sigma) for the last 45 min of heat shock. To harvest, cells were transferred into individual LoBind Eppendorf tubes if controls, or mixed in a LoBind Eppendorf tube if they were TILAC samples, and immediately placed on ice. Cells were collected by centrifugation at 750 × g and resuspended in TRIzol (Thermo Fisher).

### Transcription inhibition with flavopiridol

Cells were grown in six-well plates to ∼80% confluency. They were treated with 500 nM flavopiridol (Sigma) and 100 μM of either s^4^U or s^6^G for 2 h. After treatment, they were immediately washed in cold PBS and scraped into chilled LoBind Eppendorf tubes, at which time TILAC samples were mixed. Samples were pelleted at 1200 × g and resuspended in 500 μl of Trizol.

### Puromycin-induced ribosome dissociation and mRNA isolation

HEK293T cells were grown to ∼60% confluency and then treated with 100 μM s^4^U or s^6^G for 4 h. Plates were washed in ice-cold PBS and each plate was scraped into its own LoBind tube. Control samples were lysed in cycloheximide lysis buffer (20 mM Tris–HCl pH 7.5, 10 mM MgCl_2_, 200 mM KCl, 1% Triton, 0.2 mg/ml cycloheximide, 4 mM EDTA) and passed 10× through a 26-gauge needle. Lysate was cleared at 20 000 × g for 10 min at 4°C. Puromycin treated samples were resuspended in puromycin lysis buffer (20 mM Tris–HCl pH 7.5, 5 mM MgCl_2_, 200 mM KCl, 1% Triton, 4 mM EDTA), passed 10× through a 26-gauge needle, and cleared at 20 000 × g for 10 min at 4°C. Puromycin (VWR) was added to 2 mM, and samples were incubated on ice for 20 min, and then at 36°C for 20 min. MgCl_2_ was added up to 10 mM (23). After puromycin treatment, samples were mixed.

Following ultracentrifugation, fractions were collected into phenol:chloroform:isoamyl alcohol (Fisher Scientific). In total, one phenol extraction was performed, with two additional chloroform extractions. RNA was ethanol precipitated, and DNA removed with TurboDNase.

### Sodium arsenite stress treatment

HEK293T cells were grown in 15-cm plates and treated with 100 μM s^4^U or s^6^G for 4 h before starting the sodium arsenite treatment. Cells undergoing stress treatment were treated with 100 μM sodium arsenite for 30 min and control cells were treated with water. Plates were washed with ice-cold PBS and scraped under 1 ml of ice-cold PBS. TILAC samples were mixed, and 10% input was saved for total RNA isolation. Samples for sucrose sedimentation were lysed in cycloheximide lysis buffer (20 mM Tris–HCl pH 7.5, 10 mM MgCl_2_, 200 mM KCl, 1% Triton, 0.2 mg/ml cycloheximide, 4 mM EDTA) and passed 10× through a 26-gauge needle. Lysate was cleared at 20 000 × g for 10 min at 4°C, and then flash frozen for transportation to a collaborator for sucrose sedimentation. Samples for input sequencing were resuspended in TRIzol.

### Sucrose sedimentation and RNA isolation

Lysate was layered onto a 10–50% (w/v) sucrose gradient (20 mM HEPES pH 7.6, 100 mM KCl, 5 mM MgCl_2_, 1 mM DTT and 100 μg/ml cycloheximide). Sedimentation gradients were centrifuged for 3 h at 35 000 RPM in a Beckman SW41 rotor. All gradients were fractionated from the top down using a Biocomp Gradient Station (Biocomp Instruments) with continual monitoring of absorbance at 254 nm.

Following ultracentrifugation, fractions were ethanol precipitated, resuspended and combined into one sample. RNA was TRIzol extracted using TRIzol LS (Thermo Fisher), and DNA was removed with TurboDNase.

### Western blotting

Follow stress treatment, cells were washed with ice-cold PBS, pelleted and resuspended in RIPA buffer with protease inhibitors (Roche Complete). Lysate was incubated on ice for 10 min, passed 10× through a 26-gauge needle, and left to rotate overnight at 4°C. Lysate was combined with Laemmli's sample buffer with 2-mercaptoethanol, boiled at 95°C for 5 min before being run on a NuPAGE 4–12% Bis–Tris denaturing polyacrylamide gel (ThermoFisher) and transferred to a nitrocellulose membrane. Membrane was blocked in 1× PBS with 0.1% Tween-20 and 5% milk for 1 h at room temperature. Blots examining stress-induced proteins were incubated with primary antibodies overnight at 4°C (MCM2: Cell Signalling, 4007S; DDX5: Bethyl, A300-523A-T; α-tubulin: EMD Millipore, CP06) and with secondary antibody for 1 h at room temperature (rabbit or mouse HRP, Invitrogen). Antibody detection was performed using SuperSignal West Femto Maximum Sensitivity Substrate (Life Technologies).

### RNA sequencing and TILAC library preparation

RNA extraction and TimeLapse chemistry were performed as previously reported (21,22). Briefly, RNA was TRIzol extracted and precipitated using isopropanol supplemented with 1 mM DTT to prevent oxidation of the thiolated-bases. DNA was removed using TurboDNase, and RNA was purified using Agencourt RNAClean XP beads. TimeLapse chemistry was performed by mixing RNA with 2,2,2-trifluoroethylamine (600 mM, Fisher), EDTA (1 mM) and sodium acetate (pH 5.2, 100 mM) in water. A solution of NaIO_4_ (final concentration, 10 mM), and the reaction mixture was incubated for 1.5 h at 50°C. RNA was isolated using Agencourt RNAClean beads. RNA then went through reducing treatment to remove any excess oxidant (final concentrations, 10 mM DTT, 10 mM Tris pH 7.4, 1 mM EDTA, 100 mM NaCl) and was further purified using RNAClean beads.

Libraries were prepared using the SMARTer Stranded Total RNA-seq v2 library prep kit (Clontech). Sequencing was performed on a NovaSeq 6000 using paired-end 100 bp reads.

### Sequencing analysis

Reads were filtered for unique reads using FastUniq (24), and adaptors were removed using Cutadapt (25). Sequencing samples were aligned to both the genome and transcriptome annotations using HISAT2 (26) using default parameters and -mp4,2. Human samples were aligned to the GRCh38 genome, while Drosophila reads were aligned to the dm6 genome. Reads were further processed with Picard tools (http://broadinstitute.github.io/picard/) including FixMateInformation, SortSam, and BuildBamIndex. Reads were filtered using SAMtools to retain only those that mapped uniquely (flag: 83/163, 99/147), with MAPQ ≥2. Reads over genes were counted using HTSeq. The number of T’s, G’s, T-to-C mutations, and G-to-A mutations in each read were counted using Rsamtools (http://bioconductor.org/packages/release/bioc/html/Rsamtools.html) and a custom R script. SNPs were identified using bcftools (27) and samtools mpileup and then filtered out of mutational analysis. Tracks were made using the STAR aligner (28) (inputAlignmentsFromBam mode, outWigType bedGraph). Tracks were converted to binary format (toTDF, IGVtools) and viewed in IGV (29).

### Calculating raw mutation rates per transcript

Reads were aggregated over each gene, and the total number of T and G bases were counted, as well as the total number of T-to-C and G-to-A mutations. The mutation rate is defined as the number of mutations divided by the number of observations of the original base, e.g. T-to-C mutations divided by T observations. We did this analysis for both introns and mature transcripts, and for each type of analysis, only genes with greater than 200 reads were considered.

### DESeq2

We used DESeq2 to analyse differential expression and compare TILAC to an established statistical software. Fold-change estimates were made by using the unmixed, non-TILAC samples, including s^4^U labelled and unmixed, s^6^G labelled and unmixed, and unlabelled and unmixed samples. We used a fold-change significance cut-off of *P*_adj_ <0.05 to match the cut-off used in TILAC analyses. We also used DESeq2 to analyse transient transcriptome TimeLapse sequencing (TT-TimeLapse-seq) data from published heat shock experiments in *Drosophila* S2 cells (30), data available at Gene Expression Omnibus (GEO) under accession number GSE120220. Analyses were performed with the same fold-change significance cut-off of *P*_adj_ <0.05.

### TILAC ratios

TILAC sequencing datasets were analysed using a Bayesian–Poisson mixture model. For each forward and reverse TILAC sample in an experiment, reads were aggregated into groups which align to the same gene, were of the same mutation type (T-to-C or G-to-A) and had the same number of mutations per read. Unlabelled controls were used to determine the background expected rate of mutations from sequencing error. Induced rates of mutations for each label were modelled as a mixture of two Poisson distributions, one describing TimeLapse-induced mutations, and the other describing background mutations arising from sequencing errors. The rates of the Poisson distributions were parameterized on a log scale. The fraction of reads from each sample (experimental or control) was inferred by indexing data as whether it came from the forward or reverse experiment and using the appropriate mutational content and mixed Poisson distributions to update the log-likelihood. For example, in the forward experiment, T-to-C mutations will influence the estimate of the fraction of reads from the experimental sample, and G-to-A mutations will influence the estimate of the fraction of reads from the control sample.

The likelihood function for this model is:

$$\begin{eqnarray*} f({y_m}\;|\;{\lambda _{u,m}},\;{\lambda _{l,m}}) &=& {\theta _c}{\rm PoissonLog}\;\left( {y\;|\;\log({\lambda _{l,m}})} \right) \\ &&+ \left( {1 - {\theta _c}} \right){\rm PoissonLog}(y\;|\; {\log (\lambda _{u,m})}) \end{eqnarray*}$$

where *λ_u,m_* is the rate of mutations in unlabelled reads, *λ_l,m_* is the rate of mutations in the labelled reads, for *m* = mutation type (T-to-C or G-to-A). *y_m_* is the number of mutations per read of the given mutation type (*m*). *θ_c_* is the fraction of labelled transcripts for the condition, either experimental or control. The condition was determined by considering the label combination (forward or reverse) and the mutation type (T-to-C or G-to-A). Background mutation rates (*λ_u,m_*) were estimated in each model run by including untreated control samples (without s^4^U or s^6^G). These samples were collected at the same time as experimental samples with the exception of stress experiments, which used untreated controls from the puromycin treatment experiment.

To estimate model parameters, we used the Bayesian modelling software Stan, which implements No-U-turn Markov Chain Monte Carol (MCMC) sampling (31). For this model, we used weakly informative priors (as defined below) for expected mutation rates and fraction labelled.

Global parameter priors:

$$\begin{equation*}\log\left( {{\lambda _{u,m}}} \right)\;\sim\; {\rm Normal} \left( { - 2,\;2} \right)\end{equation*}$$

$$\begin{equation*}{\rm{log}}\left( {{\lambda _{l,m}}} \right)\; = \;{\rm{log}}({\lambda _{u,m}} + T{L_m})\end{equation*}$$

$$\begin{equation*}T{L_m}\sim\;{\rm{exp}}\left( {0.5} \right)\end{equation*}$$

$$\begin{equation*}{\rm{\;}}{I_s} = \; \begin{cases} 0 {\rm \ if\ } s\in {\rm controls}\\ 1 \ {\rm otherwise} \end{cases} \end{equation*}$$

$$\begin{equation*}g \in \;\left\{ {1,\;2,\; \ldots ,\;{n_{{\rm{genes}}}}} \right\}\end{equation*}$$

Gene-specific priors:

$$\begin{equation*}{\rm logit}({{\theta _{{\rm exp},g}}})\;\sim\;{\rm Normal}\left( {0,\;1.5} \right)\end{equation*}$$

$$\begin{equation*}{\rm logit}({\theta _{{\rm cntl},g}})\;\sim\;{\rm Normal}({0,\;1.5} )\end{equation*}$$

For read $i\; \in \;\{ {1,\;2,\; \ldots ,\;{n_g}} \}$

$$\begin{eqnarray*}&&{f_{g,m,c,s}}({y_{g,m}}\;|\;{\theta _{c,g}},\; {\lambda _{u,m}},\;{\lambda _{l,m}})\\ &&\quad= \prod_{i\; = \;1}^{{n_g}} ({I_s}{\theta_{c,g}}\;{\rm PoissonLog}\left( {{y_{i,m}}\; |\;\log\left({{\lambda _{l,m\;}}} \right)} \right)\\ &&\quad+ \left( {1 - {I_s}{\theta _{c,g}}} \right) {\rm PoissonLog}({y_{i,m}}\;|\;\log\left( {{\lambda _{u,m}}}) \right))\end{eqnarray*}$$

Additional parameter generated from estimates of other parameters:

$$\begin{equation*}{{\rm TILAC\;ratio\;}} = \frac{\theta _{{\rm exp},g}}{\theta _{{\rm cntl},g}}\end{equation*}$$

To identify significant changes in the TILAC ratio, the mean and standard deviation of the TILAC ratio posterior distribution were used to calculate a test statistic. A conservative threshold (*μ*_cutoff_ = 0.5) of significance for the magnitude of the TILAC ratio was chosen and the composite null hypothesis |mean(log_2_(TILAC ratio))| < *μ*_cutoff_ was tested rather than the null hypothesis of mean(log_2_(TILAC ratio)) = 0, following the test-statistic defined in TREAT (32). The *P*-value obtained was adjusted for multiple testing using the Benjamini–Hochberg procedure, controlling false-discovery rates (FDRs) at the 0.05 level (33).

All models converged well when using the whole dataset for analyses. Conservative filtering was applied to remove low-read count genes with highly variable coverage (read count < 200). The read count cutoff was decided based on results from simulations (see next section for details).

### Simulations

Simulations were used to assess the statistical model's accuracy and to understand the impact of sequencing depth and mutation rates on TILAC ratio estimates. To assess accuracy, a single set of TILAC experiments (i.e. one replicate of the forward and reverse labelling as well as one unlabelled sample) was simulated using TILAC ratio estimates and read counts from a real dataset (flavopiridol treated vs. no treatment). More specifically, data for 5000 transcripts were simulated with read counts being drawn from a Poisson distribution with means equal to the read count sample average from a randomly selected transcript in the TILAC dataset (data from one real transcript used for one simulated transcript), and the TILAC ratio estimate for that transcript from the analysis of the real data was used as the simulated TILAC ratio value. To assess the dynamic range of the statistical model, data from transcripts with at least 15 reads per experiment (forward label combination, reverse label combination, and no label) were used. The model used to simulate read count and mutational data in the forward labelling experiment is described below; the reverse label model is identical but with ${\theta _{\rm cntl}\;}$ and ${\theta _{\rm exp}}$ flipped; the unlabelled model only includes simulation of read counts and mutational data for reads lacking s^4^U and s^6^G:

$$\begin{equation*}{\rm No.\, of\, reads} \sim {\rm Poisson(Avg.\, real\, data\, read\, count)}\end{equation*}$$

$$\begin{equation*}{{\rm logit}(\theta_{{\rm cntl}})}\sim\frac{1}{{\left( {{\rm TILAC\;ratio}} \right)+1}}\,\;{{\rm Normal}}\left( {0,\,0.5} \right)\end{equation*}$$

$$\begin{equation*}{\theta _{{\rm exp}}}= \left({\rm TILAC{\rm{\;}}ratio} \right){\rm{*}}{\theta _{\rm cntl}}\end{equation*}$$

$$\begin{eqnarray*}&&{{\rm Labelled\, and\, unlabelled\, read\, counts }}\\ &&\quad\sim {\rm Multinomial}({\rm{No.\, of\, reads}},\;{\theta _{\rm exp}},\;{\theta _{\rm cntl}},\,1-{\theta _{\rm exp}}-{\theta _{\rm cntl}})\end{eqnarray*}$$

$$\begin{eqnarray*}&&{\rm{Number}}\,{\rm{of}}\,{\rm{Us}}\,{\rm{and}}\,{\rm{Gs}}\,{\rm{in}}\,{\rm{read}}\\ &&\quad\sim {\rm Multinomial}\left({200,\,0.25,\,0.25}\right)\end{eqnarray*}$$

$$\begin{eqnarray*}&&{\rm{Number}}\,{\rm{of}}\,{{\rm T-to-C}}\,{\rm{mutations}}\,{\rm{if}}\,{{\rm{s}}^4}{\rm{U}}\,{\rm{labelled}}\\ &&\quad\sim {\rm Binomial}\left({{\rm{Number}}\,{\rm{of}}\,{\rm{Us}},\,0.05} \right)\end{eqnarray*}$$

$$\begin{eqnarray*}&&{{\rm Number\,of\,T-to-C\,mutations\,if\,not\,}}{{\rm{s}}^4}{\rm{U\,labelled}}\\ &&\quad\sim {\rm Binomial}({{\rm Number\,of\,Us}},\,0.001)\end{eqnarray*}$$

$$\begin{eqnarray*}&&{{\rm Number\,of\,G-to-A\,mutations\,if\,}}{{\rm{s}}^6}{\rm{G\,labelled }}\\ &&\quad\sim {\rm Binomial}\left({{\rm{Number\,of\,Gs}},\,0.02} \right)\end{eqnarray*}$$

$$\begin{eqnarray*}&&{{\rm Number\,of\,G-to-A\,mutations\,if\,not\,}}{{\rm{s}}^6}{\rm{G\,labelled}}\\ &&\quad\sim {\rm Binomial}\left( {{\rm{Number\,of\,Gs}}, 0.004} \right)\end{eqnarray*}$$

Three modes of regulation were simulated using the real dataset: global upregulation/enrichment, global downregulation/depletion and equal amounts of upregulation/enrichment and downregulation/depletion. As the real dataset involved treatment of cells with a transcriptional inhibitor, the TILAC ratios in the dataset were unmodified for the global downregulation/depletion simulation. To simulate upregulation/enrichment, TILAC ratios from this dataset were multiplied by -1, and to simulate symmetric up- and downregulation, the average TILAC ratio was subtracted from each individual TILAC ratio.

A similar set of simulations was used to assess the impact of read depths and mutation rates on TILAC ratio estimate accuracy. The model used to simulate data was similar to that described above, with the following exceptions: One, a set number of read counts were simulated for each transcript. To assess the impact of read depth, a range of read counts were used (10, 50, 75, 100, 150, 200, 300, 400, 500 and 1000) with the mutation rates set at values described above. 2400 transcripts were simulated for each read count. To assess the impact of mutation rates, a set number of read counts were simulated for each transcript and a range of experimentally relevant s^6^G or s^4^U mutation rates was used (0.006, 0.008, 0.01, 0.015, 0.02, 0.025, 0.035, 0.04, 0.05 and 0.1). When testing the effect of varying the s^6^G mutation rate, the s^4^U mutation rate was set at 0.05 for all simulations, similar to that observed in real datasets. When testing the effect of varying the s^4^U mutation rate, the s^6^G mutation rate was set at 0.02, also based on the observed rate in real datasets. Variations of this simulation were analysed using 50, 100 and 1000 read counts. Two, a set TILAC ratio was simulated for each transcript, but a range of TILAC ratios were simulated in each dataset (${\rm TILAC ratio}$ = 0.01, 0.1, 0.5, 1, 1.5, 2, 10 or 100), with each TILAC ratio being used for 300 transcripts. Using a set number of read counts controlled for differences in read counts confounding the assessment of the mutation rate's impact on accuracy. A range of TILAC ratios were simulated to determine if the impacts of read depth and mutation rates were TILAC ratio dependent.

#### Gene ontology analysis

GO analysis was performed using the PANTHER database (version 16) (34) using the default parameters for statistical overrepresentation test with the annotation set GO biological process complete and using the full list of transcripts determined to be differentially regulated.

#### Gene set enrichment analysis

GSEA analysis (35) was performed on the resulting TILAC ratios using the GSEAPreranked program and comparing to the full set of gene ontology categories (c5.all.v7.3.symbols.gmt), with no collapsing of gene symbols and 1000 iterations. The statistical significance of the enrichment of helicases was assessed using the nominal rather than multiple-test adjusted *P*-value since GSEA was performed to specifically test the hypothesis of helicase enrichment in the TILAC dataset.

#### Analysis of ribosome foot-printing data

Processed data was downloaded from the Gene Expression Omnibus Portal under accession number GSE55195.

## RESULTS

### Design of an internally controlled approach to compare RNA levels

**Figure 1.**
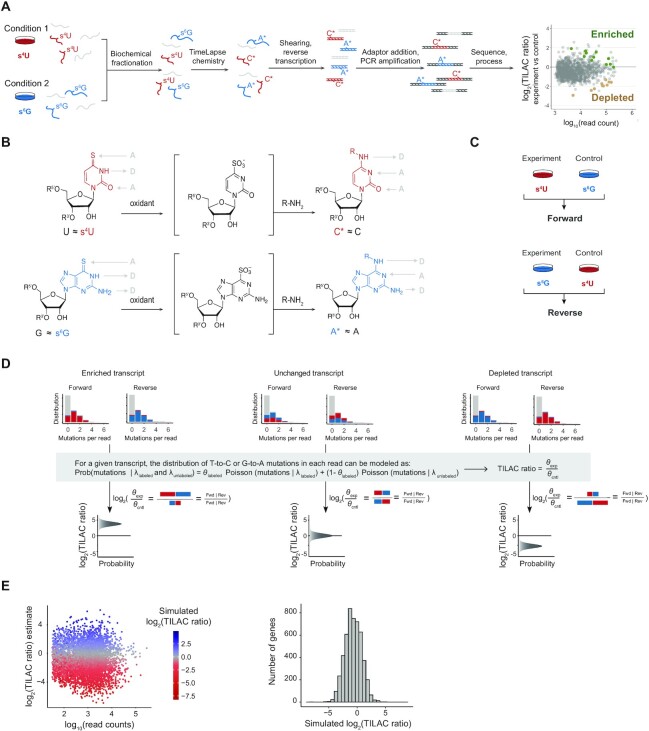
TILAC enables internal normalization of RNA sequencing experiments. (**A**) TILAC uses two metabolic labels to uniquely label two different RNA populations. Samples are combined for all downstream handling. TimeLapse chemistry induces mutations that enable differential expression analysis of internally normalized samples. (**B**) Chemical conversion of s^4^U and s^6^G to C and A analogues, respectively. (**C**) TILAC uses two reciprocal label combinations. The forward combiation treats the experimental samples with s^4^U and the control samples with s^6^G. The reverse combination treats the experimental sample with s^6^G and the control sample with s^4^U. (**D**) The TILAC Bayesian model takes into account the mutational content of each sample, and calculates a TILAC ratio, which is the log_2_ of the normalized ratio of RNA levels between the conditions. The model calculates a posterior distribution, indicating a range of plausible TILAC ratios. The posterior mean is used as the TILAC ratio estimate for all downstream analyses. (**E**) Representative analysis of simulated TILAC data. TILAC ratios and read counts were simulated according to that seen in real TILAC datasets.

To develop TILAC, we designed a protocol in which cells from different conditions were treated with either 4-thiouridine (s^4^U) or 6-thioguanosine (s^6^G). These cellular samples are mixed early in the protocol so that all downstream steps are identical and internally controlled. This ensures that the RNAs of each condition are subjected to the same handling steps, including biochemical fractionation, RNA isolation, shearing, library preparation, amplification and sequencing, which are often sources of technical variation between samples (Figure 1A). Prior to library preparation, the RNA is treated with TimeLapse chemistry to convert s^4^U to a C analogue and s^6^G to an A analogue, inducing T-to-C or G-to-A mutations in the sequencing reads (Figure 1B). To account for differences in the incorporation rates of the two different labels as well as U/G content in different transcripts, all experiments were designed to be performed with both combinations of labels. In the “forward” combination, experimental samples treated with s^4^U were mixed with control samples treated with s^6^G; in the “reverse” experiment, experimental samples treated with s^6^G were mixed with control samples treated with s^4^U (Figure 1C). Both labels exhibit minimal toxicity over the timespan of a typical experiment (Supplementary Figures S1A and S1B), as seen previously (21,22,36,37).

To interpret sequencing results from TILAC experiments, we adapted a previously validated pipeline (21) developed to estimate the fraction of labelled reads in a single sample, redesigning the analysis to estimate the ratio of labelled reads from the two different samples (Figure 1D, Materials and Methods). The mutational content of each read that maps to a given transcript in both the forward and reverse labelling experiment should allow estimation of the relative abundance of that transcript in the experimental versus control conditions. We refer to this enrichment as the TILAC ratio throughout this manuscript. This approach provides posterior distributions of parameter estimates, which describes the ratio of labelled transcripts between samples, as well as the uncertainty associated with that ratio. This information can be used to confidently identify enriched and depleted transcripts.

### Exploration of the feasibility of TILAC using simulations

To understand the dynamic range of TILAC, we simulated datasets spanning a range of read counts and mutation rates to determine the conditions over which TILAC can provide robust estimates. The analyses showed that when one of the labels has standard mutation rates of ∼5% (as is usually observed for s^4^U), the other label (s^6^G) can take on a wide range of values while maintaining confidence in the TILAC estimates. The same is true when using a fixed mutation rate of ∼2% (as is usually observed for s^6^G). Under this simulation, s^4^U can take on a wide range of values while maintaining confidence in the estimates. Due to the lower fixed s^6^G rate (2% versus 5%), estimate accuracy is slightly lower than for equivalent simulated transcripts with a fixed s^4^U mutation rate (Supplementary Figure S2A). In contrast to mutation rates, read counts had a larger effect on the ability to confidently calculate TILAC ratios. TILAC ratios from transcripts with greater than 200 reads were determined with high confidence across TILAC ratios ranging from 0.1 to 100 (−1 to 2 on a log_10_ scale, Supplementary Figure S2B). We used these results to set a threshold of 200 reads mapped in at least two samples as a minimum read count below which a transcript is removed from further analysis. To further validate our analyses, we simulated TILAC sequencing data with similar distributions of read counts and mutations as real data, over a range of TILAC ratios. Three instances of regulation were tested: (i) nearly equal amounts of upregulation/enrichment and downregulation/depletion of RNA levels in the experimental condition, (ii) the experimental condition contains more RNA due to upregulation or enrichment or (iii) the experimental condition contains less RNA due to downregulation or depletion. In each case, the TILAC model was able to accurately estimate the true TILAC ratios and capture the simulated mode of regulation (Figure 1E, Supplemental Figures S2C and D).

### TILAC can identify global differences in RNA levels in experimental samples

We next sought to validate TILAC using experimental samples by comparing RNA from cells treated with an inhibitor of RNA Polymerase II (Pol II) to those from control cells and testing whether TILAC could detect the relative loss of transcripts. We treated cells with flavopiridol for 2 h (9), a CDK9 inhibitor that blocks transcription of Pol II-dependent genes but does not affect the transcription of genes transcribed by Pol I and Pol III, such as *RN7SL1*. We performed the forward and reverse TILAC experiments in duplicate (for a total of four samples).

We first examined the total read counts from these samples to ascertain if TILAC labelling and chemical treatment negatively influenced the RNA-seq data. The counts from replicate and different mixture combinations were all highly correlated (Pearson's *r* = 0.97–0.98), demonstrating minimal impact of the different labelling schemes on total RNA levels (Supplementary Figure S3A). This is consistent with previous work which demonstrated that metabolic labelling under similar conditions can be performed with minimal toxicity to the cells and that nucleotide recoding does not negatively impact RNA-seq analyses (21,22,36,37). We also compared the read counts from unmixed, individually labelled control samples, which also showed good agreement (Pearson's *r* = 0.97-0.98), further establishing the minimal impact of the labels on RNA levels when performing TILAC (Supplementary Figure S3B).

**Figure 2.**
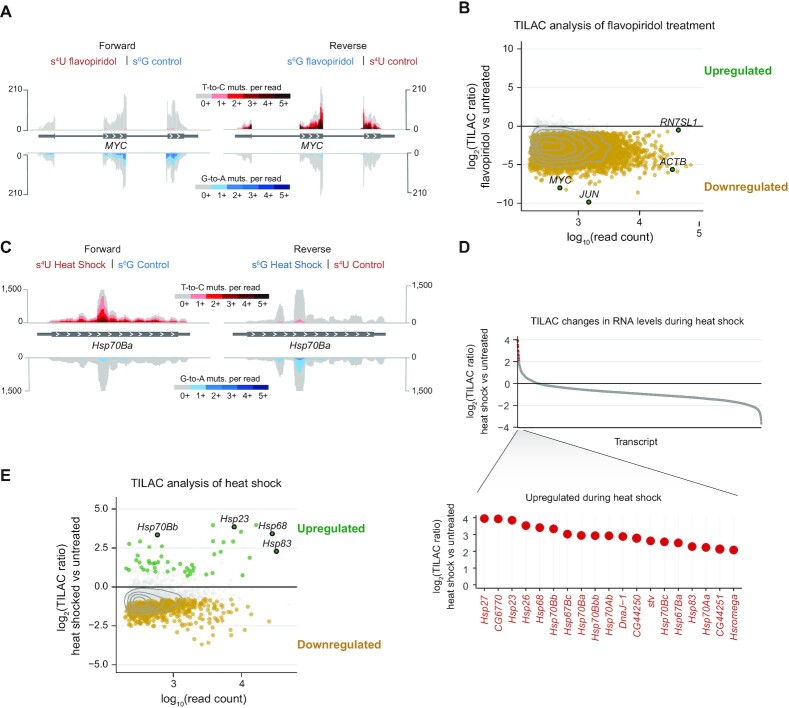
TILAC captures transcriptional changes in two different systems. (**A**) Sequencing tracks with the forward experiment on the left and the reverse experiment on the right. Gray signal indicates total reads. Above, reads with T-to-C mutations are indicated in red. Below, G-to-A mutations are indicated in blue. (**B**) TILAC analysis of transcriptional inhibition with flavopiridol (two forward replicates and two reverse replicates, four total, were used in this analysis). (**C**) Tracks showing enrichment of T-to-C and G-to-A mutations in the heat shock responsive gene *Hsp70Ba*. (**D**) (Upper) All transcripts analyzed by TILAC, ordered by their log_2_(TILAC ratio). (Lower) Transcripts that are more than 4-fold upregulated according to TILAC are known heat-shock proteins. (**E**) TILAC analysis of differential gene expression upon heat shock (two each forward and reverse replicates).

We next examined the T-to-C and G-to-A mutation rates in each sample. Intronic mutation rates were calculated for each sample. As expected, there was enrichment of T-to-C mutations only in samples treated with s^4^U (Supplementary Figure S3C), and enrichment of G-to-A mutations only in samples treated with s^6^G (Supplementary Figure S3D). The untreated samples showed low levels of background mutations for both samples. We note that s^6^G treatment at the same concentration as s^4^U yields fewer mutations, as previously observed (22), and as we also find in our unmixed control samples (Supplementary Figure S3). As noted above, simulations had established this lower rate was unlikely to adversely influence TILAC analyses. When transcription is inhibited with flavopiridol in s^4^U-labelled samples and mixed with control cells labelled with s^6^G, the sequencing results for all exonic and intronic reads are enriched for reads with G-to-A mutations, but not T-to-C mutations. The opposite is true for the reverse experiment, indicating that this approach accurately captures the inhibition of transcription in the experimental sample (Figure 2A). The overall mutation rates calculated from both exonic and intronic reads are reproducible between replicates and also agree between mixed and unmixed samples (Supplementary Figure S4). These results demonstrate that the RNA metabolic labels are incorporated as expected and do not interfere with RNA metabolism and transcriptome analyses.

Application of the TILAC analysis pipeline to all exonic and intronic reads revealed that RNA levels were decreased upon inhibition of RNA Pol II compared to control samples, as expected. Indeed, the analysis of the TILAC ratios showed a significant drop for the majority of highly expressed transcripts (6437 of 7621 transcripts) (Figure 2B). These TILAC ratios were determined using the statistical modelling approach described above (Supplemental Figure S5). We also note that the highly expressed Pol III transcript *RN7SL1* has a log_2_(TILAC ratio) near 0, indicating it is largely unchanged, as would be expected given that flavopiridol treatment does not lead to substantial inhibition of Pol III (Supplementary Table S2). These results were corroborated using standard statistical analysis software, which also identified downregulation of Pol II transcripts, but not of Pol I or III transcripts. We note that TILAC’s internal normalization captures global downregulation of Pol II transcripts compared to an unmixed and unspiked RNA-seq analysis (Supplementary Figure S6A, Supplementary Table S1).

### Transcriptome changes upon heat stress are detected by TILAC

Next, we tested whether TILAC could accurately identify the upregulation of a few specific transcripts as well as the global transcriptional downregulation that is associated with heat shock. During the heat shock response in *Drosophila*, some heat shock genes are induced, while transcription from the rest of the genome is dramatically reduced (30,38–40). We treated *Drosophila* S2 cells under heat shock conditions (37°C for an hour compared to 27°C for control cells) and treated the cells with either s^4^U or s^6^G for the last 45 min of heat treatment. Examination of the mutations in the browser tracks revealed that the heat shock transcripts were induced and contained mutations reflective of their metabolic labelling treatment (Figure 2C). Global analysis by TILAC demonstrated the broad downregulation of most transcripts and the upregulation of 49 transcripts (Figure 2E, Supplementary Table S3). These upregulated transcripts are highly enriched for those previously identified as heat shock transcripts (Figure 2D; PANTHER analysis- cellular response to unfolded protein: fold enrichment 82.16, FDR 4.9 × 10^−15^ and cellular response to heat: fold enrichment 83, FDR 4.3×10^−12^). We compared these TILAC results to standard, unmixed RNA-seq analyses under the same conditions to confirm our results. This statistical analysis also identified the same highly upregulated heat shock transcripts, as well as a number of downregulated transcripts (Supplementary Figure S6B, Supplementary Table S1). We note that TILAC is able to identify many transcripts (696) that are transcriptionally downregulated compared to an unmixed and unspiked RNA-seq analysis due to the internal normalization inherent to the TILAC experimental design. Comparison of our TILAC results to previous transient-transcriptome-sequencing data (TT-TimeLapse-seq) confirms that the transcripts identified as down regulated by TILAC are transcriptionally repressed upon heat shock (including all 625 present in all data sets that were missed by conventional RNA-seq analyses, (Supplementary Table S4, Supplementary Figure S6) (30).

### TILAC reveals selective translation during stress

**Figure 3.**
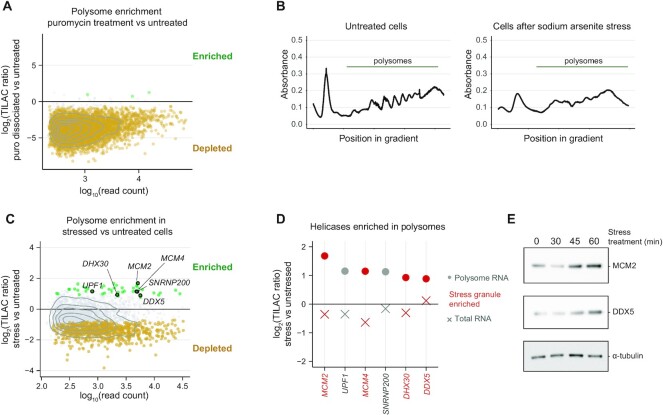
TILAC reveals translational upregulation of helicases during sodium arsenite stress. (**A**) TILAC analysis of what is depleted from polysomes between puromycin-treated compared to untreated cells (two each forward and reverse replicates). (**B**) Absorbance traces showing polysomes in untreated 293T cells that are significantly depleted in stressed cells. (**C**) TILAC analysis of transcripts in polysomes following sodium arsenite stress (one forward sample and one reverse sample). (**D**) Six helicase-encoding transcripts are enriched in polysomes during stress but are not transcriptionally upregulated. Highlighted in red are transcripts whose protein products are enriched in stress granules (48). (**E**) Cellular MCM2 and DDX5 protein levels are elevated upon stress treatment.

Having established that TILAC can provide internally normalized analyses of RNA levels in unfractionated samples, we next explored the extent to which TILAC can provide internal controls for experiments that involve complex biochemical steps such as cellular fractionation. As a stringent test of the TILAC approach, we asked if TILAC could reveal ribosome-associated RNA under stress conditions, thereby informing on differences in translation regulation of specific RNA transcripts. To identify mRNAs that are being translated, ribosomes and their associated RNA can be isolated by fractionation through a sucrose gradient (41, 42). The fractions of the gradient that contain the polysome peaks, defined as having two or more ribosomes per mRNA, are collected and the associated RNA is sequenced. We hypothesized that TILAC could address two challenges in these experiments: (i) normalization and (ii) internal controls for variability so that the comparisons are one-to-one despite large numbers of handling steps. To study RNA recruited to polysomes, we first assessed if we could detect puromycin-induced dissociation of actively translating ribosomes from their mRNA. Comparing bulk polysome profiles from cells treated with puromycin to untreated controls showed polysome peaks were visible in untreated cell lysate, but they decreased when mixed with ribosome-dissociated lysate (Supplemental Figure S7A). We collected fractions associated with peaks containing two or more ribosomes per transcript and performed TILAC (Supplementary Figure S7A). As expected, our statistical analysis indicated that the vast majority of transcripts were significantly depleted in puromycin-treated samples (5341 depleted out of 5439 transcripts) (Figure 3A), indicating that TILAC is suitable to study changes in RNA association with ribosomes (Supplementary Table S5).

Having validated TILAC’s performance in control experiments involving ribosome fractionation, we employed the protocol to investigate translational changes during arsenite stress. Sodium arsenite stress activates the cell's integrated stress response, a set of molecular changes that help the cell survive many different types of stress and involves the total reprogramming of translation and the formation of stress granules (43). Human cells were treated for 4 hours with metabolic labels prior to 30 min of sodium arsenite stress (44–46). Consistent with the expected downregulation of translation, defined polysome peaks were disrupted in stressed cells (Figure 3B). The data from the puromycin experiment, as well as a puromycin comparison performed under stress conditions, were used to identify potentially contaminating background from other RNP’s that could be in the polysome fractions (Supplemental Figure S7B, Supplementary Tables S6 and S7). These potential background RNAs were eliminated from analysis of stressed samples, revealing 42 transcripts enriched in polysomes during stress (Figure 3C). These transcripts are newly identified compared to a previous study of sodium arsenite stress, likely due to differences in study design and technique between ribosome foot-printing and polysome profiling (Supplementary Figure S7C) (45). While mRNAs with long ORFs are known to take longer to exit translation (47), the ORF sizes of these 42 transcripts ranged from 2 to 105 kb with no apparent length bias in retained transcripts compared to the whole transcriptome (Supplementary Figure S7D, Supplementary Table S8). To test whether these transcripts were enriched specifically in the polysome fraction, we also performed TILAC on the input RNA prior to polysome fractionation to look for transcriptional upregulation and saw no transcriptional regulation of these transcripts (Supplemental Figure S7E, Supplementary Table S9). This supports the conclusion that these transcripts are translationally regulated.

Interestingly, six of the transcripts that were enriched in the polysome fraction during stress encode helicases (*MCM2*, *UPF1*, *MCM4*, *SNRNP200*, *DHX30*, *DDX5*; GSEA nominal *P*-value approaching 0), and four of those helicases are known to localize to stress granules (Figure 3D). This finding is intriguing because a previous study indicated that knock down of one of the proteins (MCM2) causes stress granules to dissolve faster during stress (48). Based on the polysome enrichment observed by TILAC, we hypothesized that MCM2 and DDX5 are translationally upregulated. We observed a time-dependent increase in protein levels of these helicases during stress (Figure 3E), supporting our conclusion that TILAC has revealed stress-specific regulation of translation of these helicase transcripts.

## DISCUSSION

TILAC provides a new approach to internally control comparisons of RNA levels between samples. Comparing RNA levels using RNA-seq experiments can be challenging due to variability in biochemical enrichment and handling, and difficulties normalizing across samples. We anticipate that TILAC will be a powerful tool in a wide range of applications, from studying RNA-protein interactions, to gene expression, and synthetic biology. As we have demonstrated using transcriptional inhibition and heat stress, TILAC provides internal controls to account for both variance and normalization in standard RNA-seq experiments, thereby illuminating global changes in RNA levels as well as specifically regulated transcripts, such as those upregulated in the heat shock response (Figure 2). We have shown that TILAC can be extended to fractionations, where it is most necessary to control for differences in handling (Figure 3, Supplementary Figure S8). TILAC succeeded in identifying depletion of transcripts in a polysome fractionation experiment upon puromycin treatment.

TILAC is a powerful method but has certain limitations. First, TILAC is only compatible with systems that are amenable to metabolic labelling. Furthermore, the observed changes are only reflective of the transcripts made after the start of the metabolic labelling step. Longer metabolic labelling treatments are preferred when the experiment is designed to detect changes in steady-state levels of the transcriptome. On the other hand, we anticipate that shorter and more targeted treatments with metabolic labels could be used to examine acute changes in the newly made transcript pool after a perturbation. Longer treatments with s^6^G can induce cell toxicity (Supplemental Figure S1), and tolerance to different treatment times and concentrations vary between cell lines. The impact of labelling can be determined by monitoring cell health with and without nucleoside treatment. As with any metabolic labelling experiment, RNA-seq analysis of nucleoside-treated versus untreated samples can be used to determine the impact of labelling on the transcriptome. Experimental designs that involve shorter treatment times can help minimize concerns related to nucleoside toxicity. While s^6^G treatment leads to lower mutation rates than s^4^U, simulations and experimental data demonstrate that the forward/reverse experimental design remains robust and the ability of TILAC to accurately identify changes in transcript levels is high (Figure 1). While TILAC ratio estimates are not very sensitive to low s^6^G-induced mutations rates, we find the accuracy of TILAC ratios is sensitive to read depth. Like other RNA-seq based methods, changes in levels of lowly expressed transcripts (<200 reads) can be difficult to estimate accurately with TILAC and we therefore limited our analyses to the thousands of transcripts that exceed this threshold in each experiment (Figure 1).

In this report, we performed TILAC by chemically recoding nucleotides using TimeLapse chemistry because it is compatible with s^4^U and s^6^G under the exact same chemical conditions. TimeLapse chemistry does not require treating RNA under basic conditions, nor does it require the use of heavy metals. Nonetheless, we note that TILAC is in principle compatible with other chemistries (17–19) and will only become more powerful with further advances in metabolic labelling and nucleotide recoding methodologies.

TILAC revealed a new set of transcripts that are polysome enriched during stress. Among these transcripts is *MCM2*, which regulates stress granules in both yeast and humans. Mutations in another transcript in this set, *DHX30*, leads the protein to localize to stress granules and causes translational repression (49), and are associated with developmental delays and intellectual disability. Our result builds on the field's increasing appreciation for the role of ATP-dependent helicases during the cellular stress response to prevent toxic RNA aggregation (50), demonstrating for the first time the translational regulation of helicases involved in this stress response. These results showcase the power of TILAC to uncover nuanced transcriptional and translational responses and paves the way for future studies on the specific translation of these transcripts during stress.

## DATA AVAILABILITY

The pipeline for analysing sequencing data is available at https://bitbucket.org/mattsimon9/timelapse_pipeline/src/master/.

Sequencing data that support the findings of this study have been deposited in the Gene Expression Omnibus (GEO) under accession number GSE168716.

## Supplementary Material

gkac693_Supplemental_Files

## Notes

Present address: Lea Kiefer, Department of Neurology, UCSF, San Francisco, CA 94143, USA.
