# Supplementary material for: Internally controlled RNA sequencing comparisons using nucleoside recoding chemistry
Source: Nucleic Acids Res. 2022 Aug 26;50(19):e110. doi: 10.1093/nar/gkac693 (PMC9638901; doi:10.1093/nar/gkac693)
Supplement: gkac693_Supplemental_Files [file gkac693_supplemental_files.zip › TILAC_Supplementary_Data.docx]

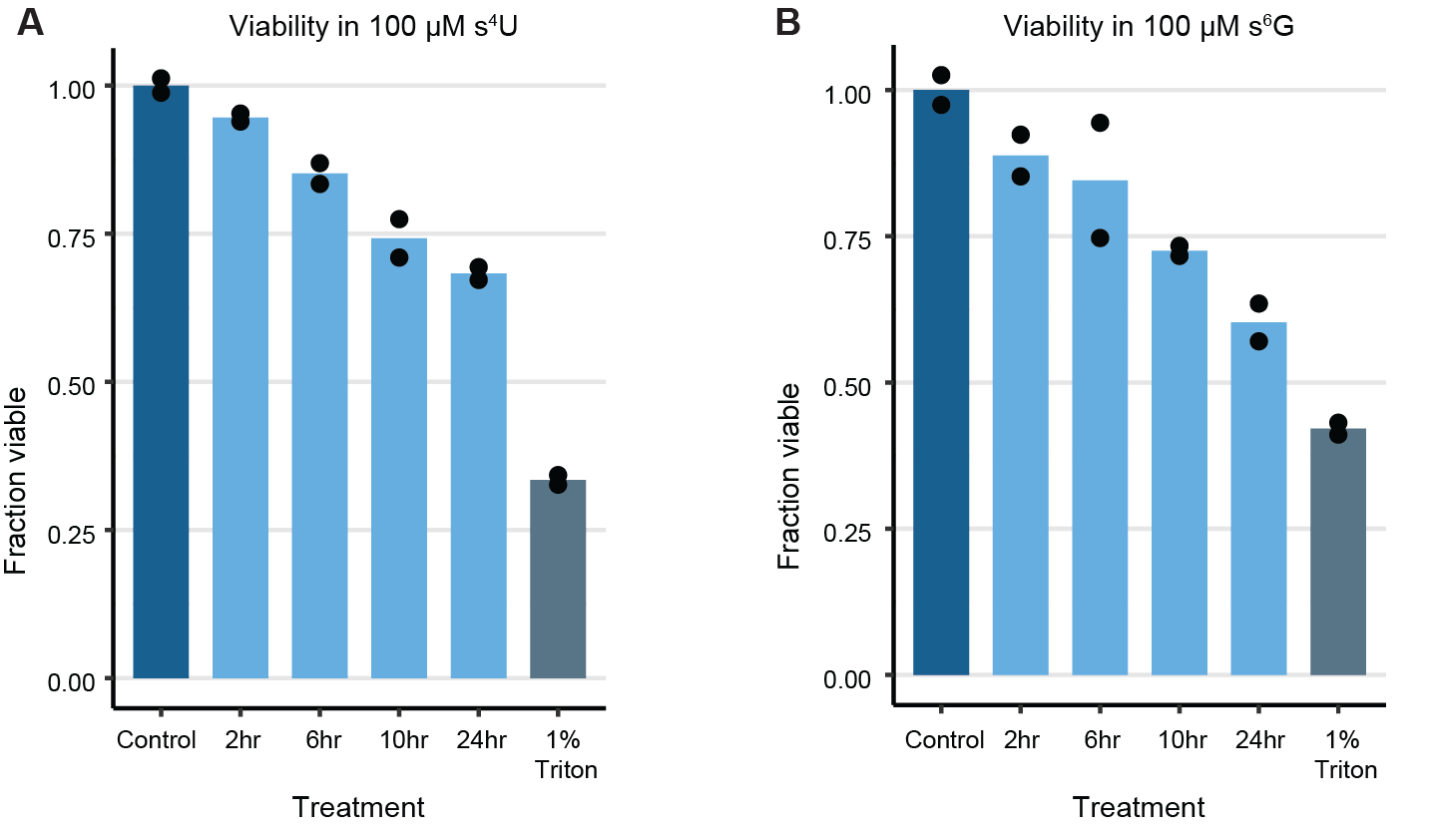


**Supplementary Figure S1.** Viability of 293T cells while being treated with either nucleotide analogue, s^4^U or s^6^G. (**A**) Cells were treated with s^4^U for the indicated time, or with 1% Triton as a negative control. The positive control was treated with water vehicle. (**B**) Cells were treated with s^6^G for the indicated time, or with 1% Triton as a negative control. The positive control was treated with DMSO vehicle.


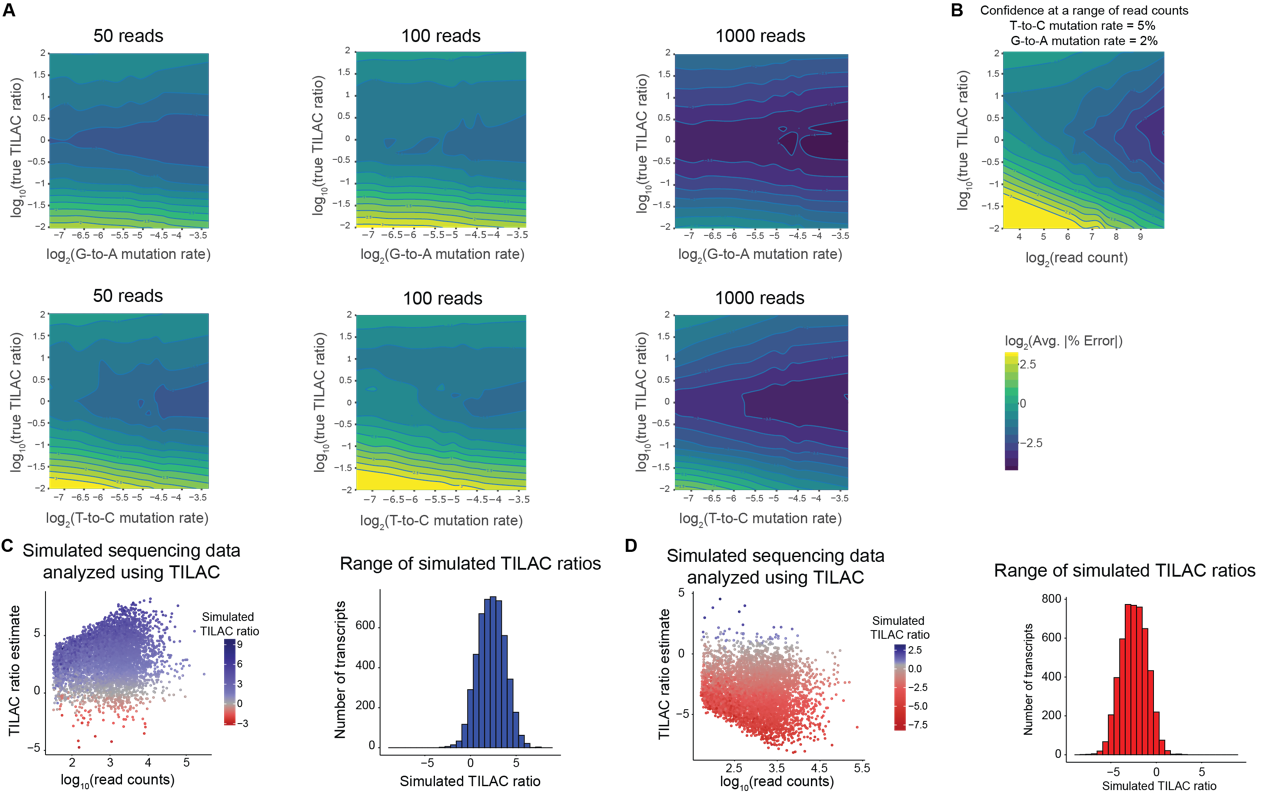


**Supplementary Figure S2.** Validation of TILAC through simulation. (**A**) The accuracy of the TILAC ratio estimates across a range of G-to-A and T-to-C mutation rates, for the number of reads-per-transcript indicated above the graphs. The colored contours indicate the log_2_ transformed average absolute percent error (averaged over 300 simulated transcripts for each datapoint) at a given combination of true TILAC ratio and G-to-A or T-to-C mutation rate. When testing the effect of varying the G-to-A mutation rate, a fixed T-to-C mutation rate of 5% (typical for s^4^U labeling and nucleotide recoding) was simulated. When testing the effect of varying the T-to-C mutation rate, a fixed G-to-A mutation rate of 2% (typical for s^6^G labeling and nucleotide recoding) was simulated. The legend for all contour plots in **A** and **B** is shown below the plot in **B**. (**B**) The accuracy of TILAC ratio estimates across a range of transcript-specific read counts, for datasets simulated with a 5% T-to-C mutation rate and a 2% G-to-A mutation rate. (**C, D**) TILAC analysis of simulated TILAC data. TILAC ratios and read counts were simulated according to that seen in real TILAC datasets. MA plots are colored by the true simulated TILAC ratio. Histograms depict the distribution of TILAC ratios simulated. (**C**) Simulated global upregulation is accurately identified by TILAC. (**D**) Simulated global downregulation is accurately identified by TILAC.


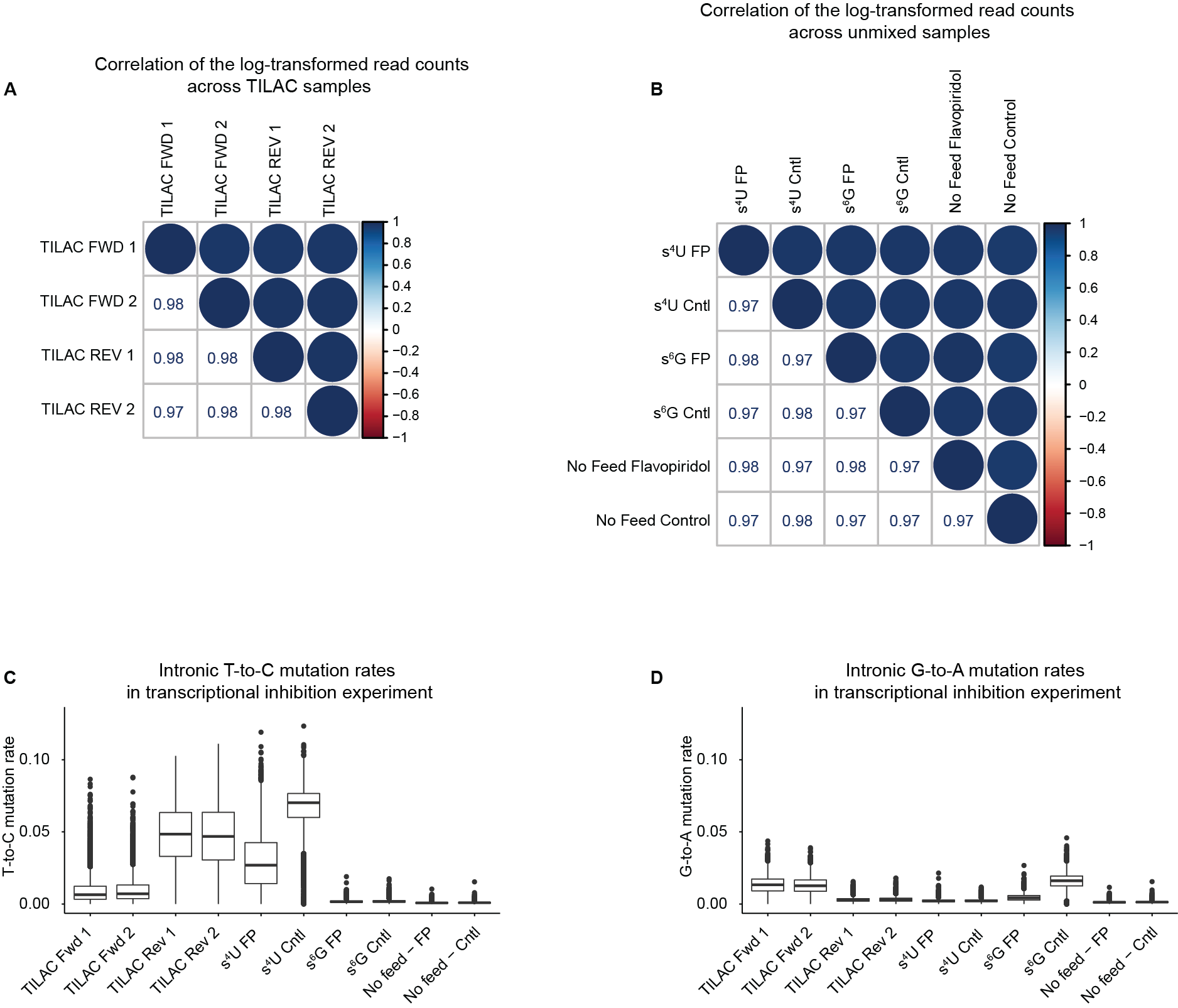


**Supplementary Figure S3**. Evaluating TILAC in flavopiridol-induced transcription inhibition. (**A**) Correlation of log-transformed read counts between TILAC samples, indicating that mixing two different labeled populations does not significantly affect the transcriptome. Strong correlation is observed between all samples, with Pearson’s r values indicated below the diagonal. (**B**) Correlation of the RNA-seq log-transformed read counts between single-label and unfed samples, indicating that labeling has negligible effects of the transcriptome. (**C,D**) Mutation rates per gene are calculated and compared across feed and mixing conditions. (**C**) T-to-C mutation rates. In the forward TILAC experiment, the s^4^U-treated samples are flavopiridol treated. In the reverse TILAC experiment, the s^4^U-treated samples are the control samples. (**D**) G-to-A mutation rates. In the forward TILAC experiment, the control samples are s^6^G-treated, and in the reverse TILAC experiment the flavopiridol treated samples are s^6^G-treated.


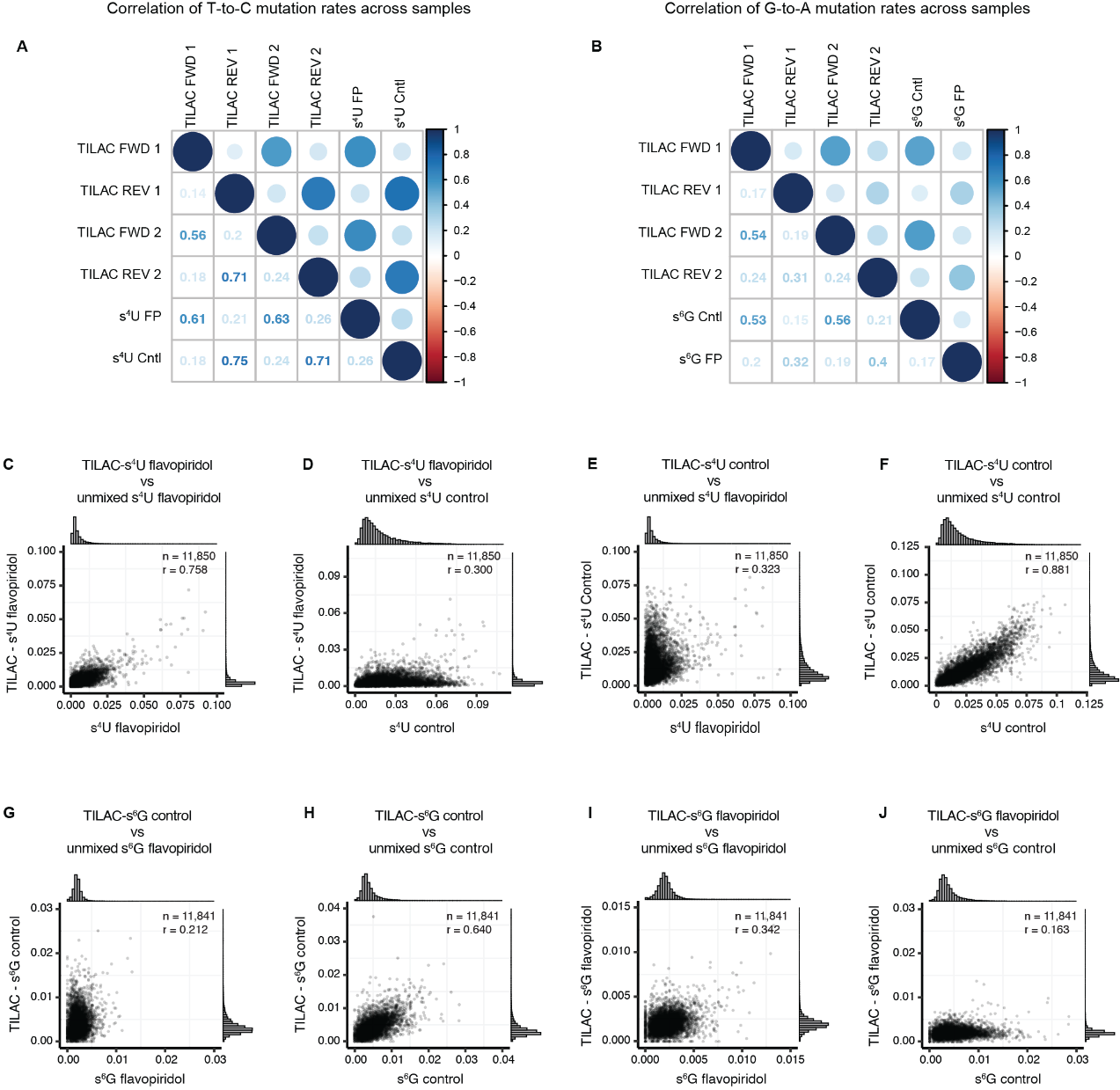


**Supplementary Figure S4.** Comparison of overall mutation rates, calculated from both intronic and exonic reads, between TILAC and unmixed samples. (**A**) Correlation of unprocessed T-to-C mutation rates between TILAC samples and singly-labeled samples, indicating agreement in labeling between conditions. (**B**) Correlation of unprocessed G-to-A mutation rates between TILAC samples and singly-labeled samples. (**C, D**) The T-to-C mutation rate of all reads in the forward TILAC experiment is compared to the mutation rate of the (**C**) unmixed, s^4^U-treated and flavopiridol treated sample and (**D**) the unmixed, s^4^U-treated and control sample. (**E, F**) The T-to-C mutation rate of all reads in the reverse TILAC experiment is compared to the mutation rate of (**E**) the unmixed, s^4^U-treated and flavopiridol treated sample and (**F**) the unmixed, s^4^U-treated and control sample. (**G, H**) The G-to-A mutation rate of all reads in the forward TILAC experiment is compared to the mutation rate of the (**G**) unmixed, s^6^G-treated and flavopiridol treated sample and (**H**) the unmixed, s^6^G-treated and control sample (**I, J**) The G-to-A mutation rate of all reads in the reverse TILAC experiment is compared to the mutation rate of the (**I**) unmixed, s^6^G-fed and flavopiridol treated sample and (**J**) the unmixed, s^6^G-treated and control sample.


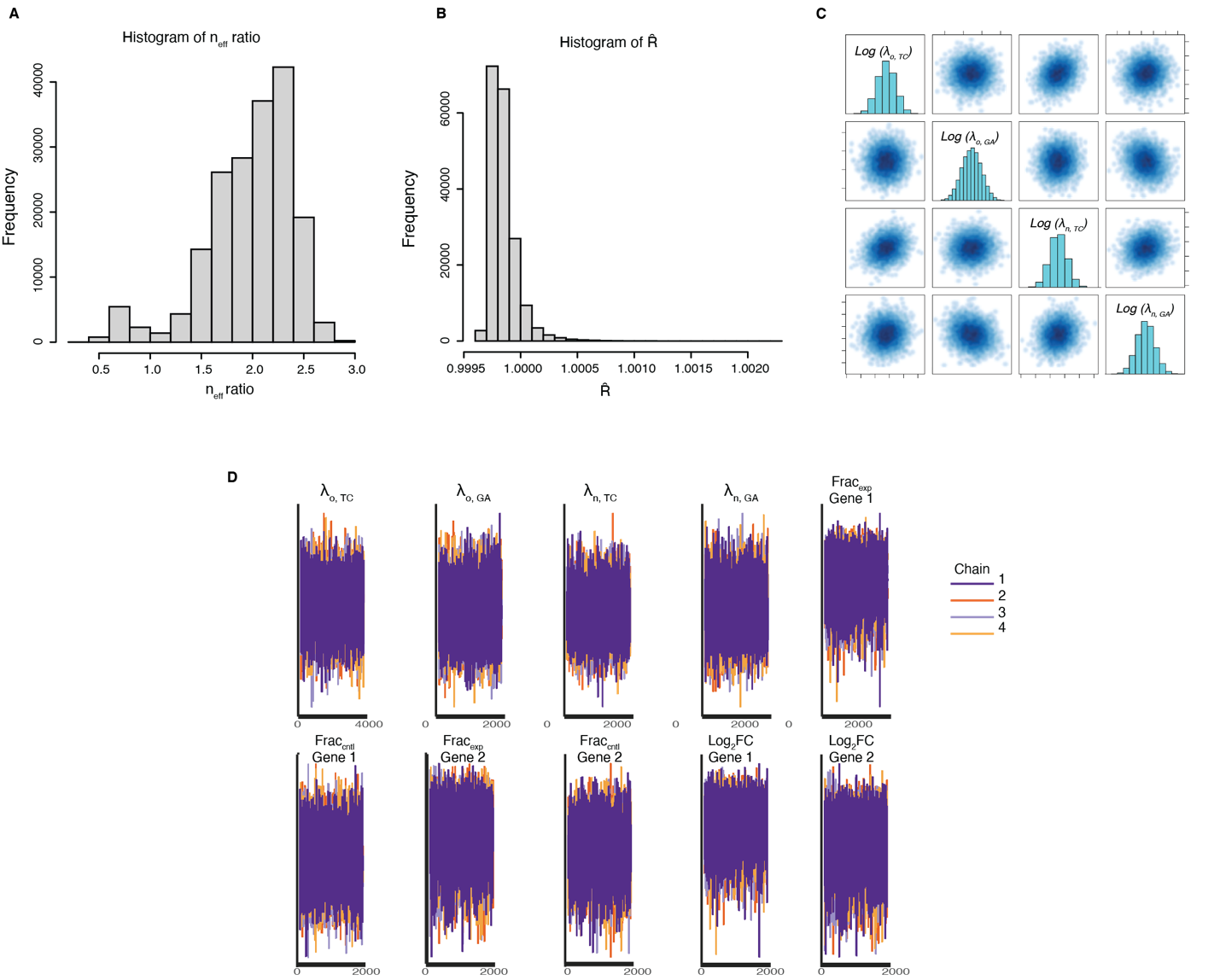


**Supplementary Figure S5.** TILAC model validation analyzing flavopiridol-induced transcriptional inhibition.­ (**A**) n*_eff_* ratios are predominantly above 1, indicating the model sampled efficiently. (**B**) ­­­ $\hat{R}$ values are less than 1.1, indicating model convergence. (**C**) Uncorrelated normally distributed pairs plots indicate independence of estimates. (**D**) Chains demonstrate mixing and minimal Markov chain autocorrelation.


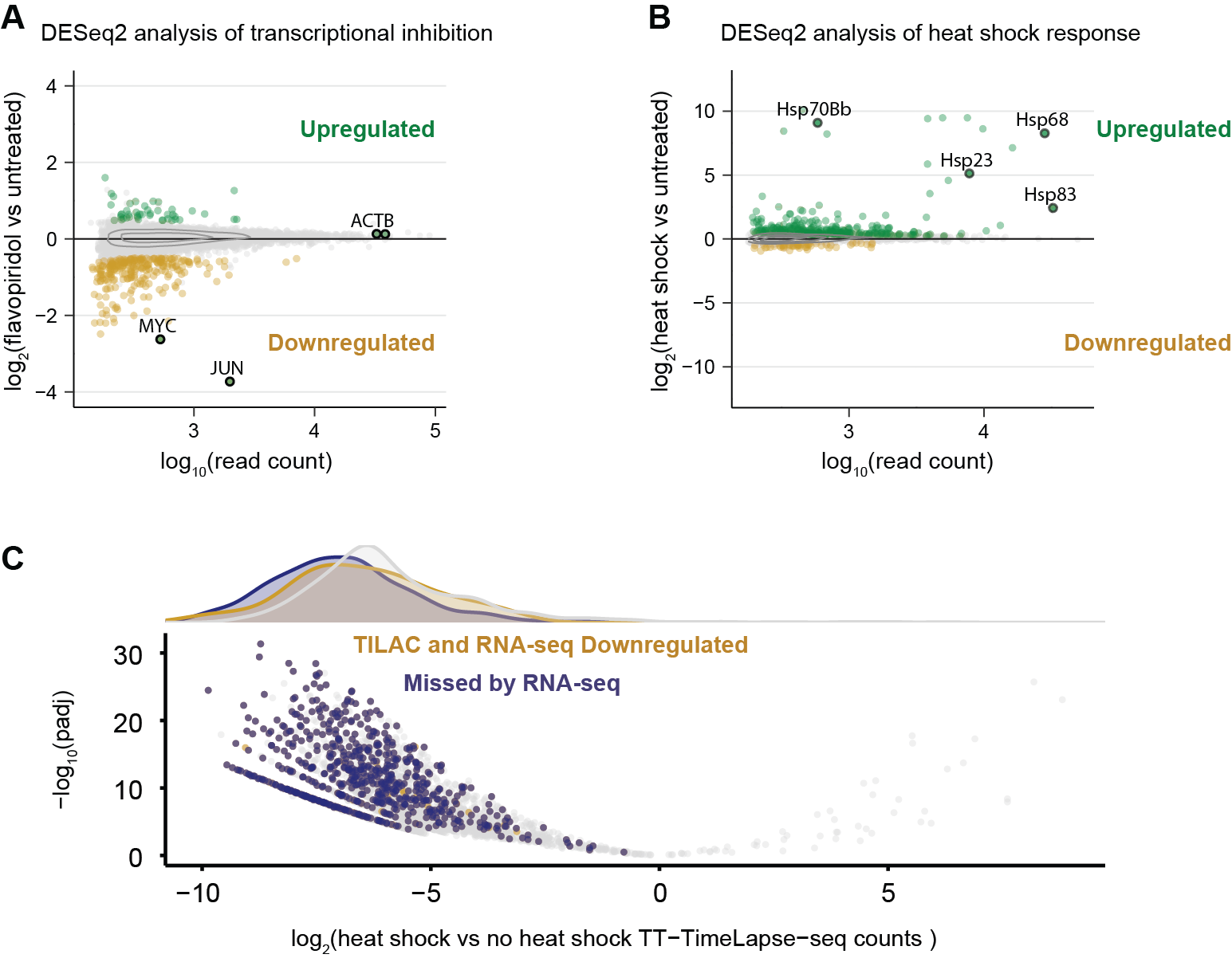


**Supplementary Figure S6.** DESeq2 analysis of differential expression of RNA sequencing data. **(A**) DESeq2 analysis of the unmixed samples, comparing flavopiridol treated to untreated. Contour lines indicate the density of data. **(B**) DESeq2 analysis of the upregulation of heat shock responsive transcripts. **(C**) All transcripts TILAC identifies as downregulated have a negative TT-TimeLapse-seq fold change. 652 out of 655 of these TT-TimeLapse-seq fold changes are significant (FDR < 0.05). For TT-TimeLapse-seq data, unnormalized read counts were passed to DESeq2 for statistical analysis, with all normalization factors set to one. Many of the transcripts that TILAC identifies as downregulated are missed by DESeq2, shown in blue are validated by their negative log2 fold change in TT-TimeLapse-seq.


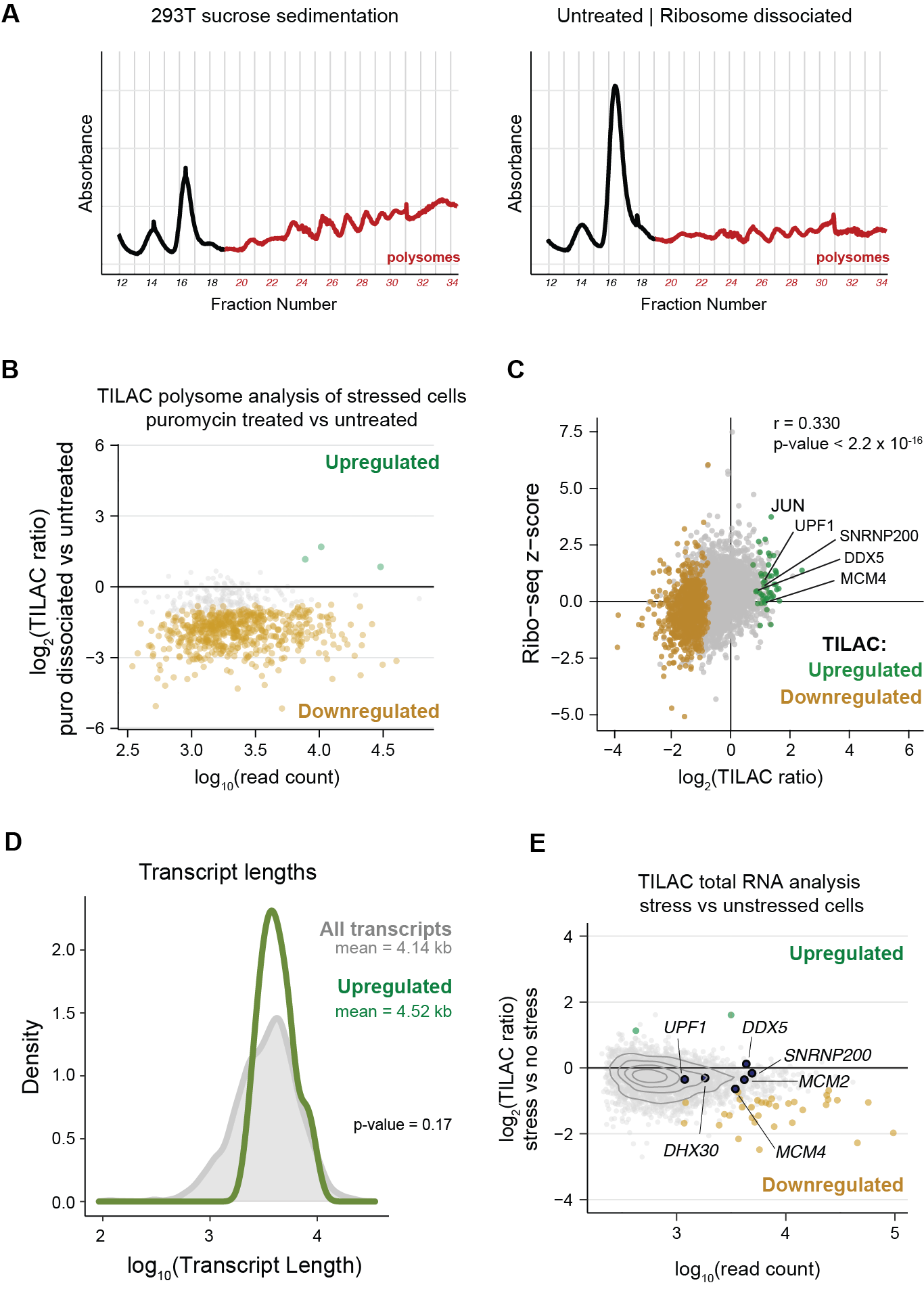


**Supplementary Figure S7.** Controls for the TILAC experiment examining cellular response to stress. (**A**) Absorbance traces showing polysomes in untreated 293T cells, that are reduced when mixed with puromycin-treated cells. Polysome fractions are indicated in red. (**B**) TILAC analysis of transcripts following puromycin-induced ribosome dissociation in stress-treated cell lysate. (**C**) Comparison of changes in translation as measured by polysome profiling (TILAC) or ribosome foot printing (Ribo-seq). Similar trends are captured with both methods, notably the enrichment on ribosomes of the helicase transcripts *UPF1*, *SNRN200*, *DDX5*, and *MCM4*. (**D**) RNAs retained on ribosomes have a similar length distribution to all cellular transcripts. (**E**) RNA-seq analysis shows few transcriptional changes occurring during stress. Helicase transcripts are highlighted and do not experience significant changes in total RNA levels.


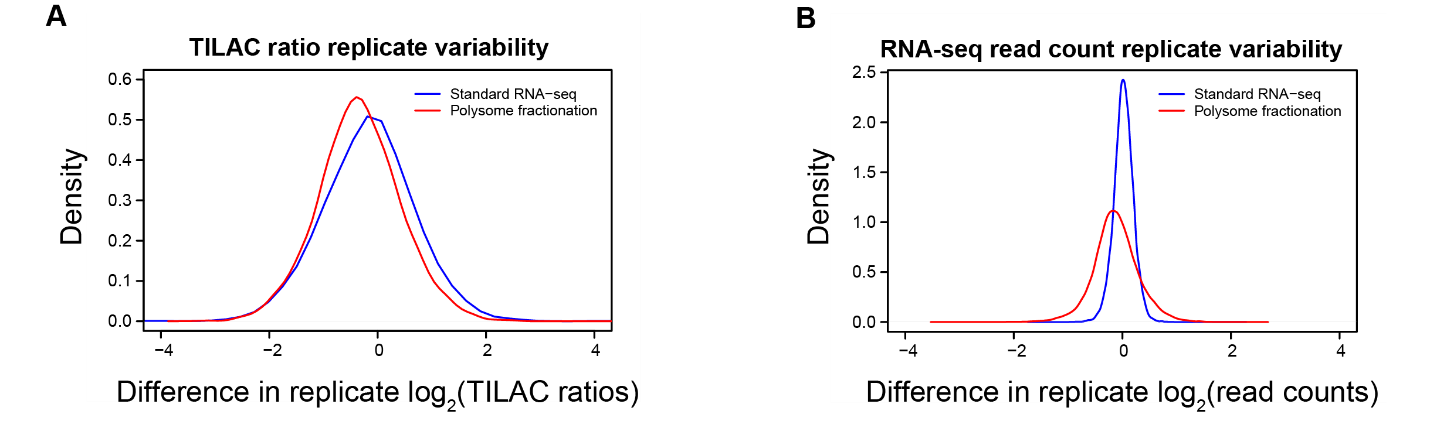


**Supplementary Figure S8.** Comparison of TILAC ratio and RNA-seq read count replicate variability. **(A)** Distribution of the difference between TILAC ratio estimates in two replicates (one replicate includes one sample of each label combination) in either the flavopiridol vs. untreated experiment (Standard RNA-seq) or the polysome fraction of the puromycin vs. untreated experiment (Polysome fractionation). **(B)** Distribution of the difference between log2 transformed normalized read counts in the same experiments as in (**A).** While the variability in the TILAC ratio is nearly identical for both protocols, normalized read counts show greater variability in the fractionation experiment. RNA-seq read counts were normalized using transcripts that had a TILAC ratio close to zero in both replicates of the TILAC analysis and thus should be unchanged between samples.
